# Efficacy and safety of sorafenib plus vitamin K treatment for hepatocellular carcinoma: A phase II, randomized study

**DOI:** 10.1002/cam4.3674

**Published:** 2021-01-22

**Authors:** Yoshimichi Haruna, Takayuki Yakushijin, Seiichi Kawamoto

**Affiliations:** ^1^ Departments of Laboratory Medicine Osaka General Medical Center Osaka Japan; ^2^ Liver Cancer Center Osaka General Medical Center Osaka Japan; ^3^ Gastroenterology and Hepatology Osaka General Medical Center Osaka Japan; ^4^ Diagnostic Imaging Osaka General Medical Center Osaka Japan

**Keywords:** hepatocellular carcinoma, prospective study, prothrombin, sorafenib, vitamin K

## Abstract

The previous retrospective study suggested that dosing vitamin K may enhance the anticancer action of sorafenib against hepatocellular carcinoma. To confirm it, we performed a phase II, randomized, open‐label study. Patients with hepatocellular carcinoma were randomly assigned to receive sorafenib + vitamin K2 (menatetrenone, 45 mg daily, orally) or sorafenib only. Between 1 May 2012 and 1 May 2016, 68 patients were screened. Forty‐four eligible patients were assigned at a 1:1 ratio to each cohort. The objective response rate in the vitamin K‐dosed group was significantly higher than that in the sorafenib only group (27.3% vs 4.5%, respectively; *p* = 0.039). The median time of progression‐free survival was significantly extended in the vitamin K‐dosed group compared with the sorafenib only group (4.9 months vs 2.7 months, respectively; hazard ratio (HR), 0.44; 95% confidence interval (CI): 0.21–0.89; *p* = 0.018). Although there was no significant difference between the two groups in the median time of overall survival, patients in the vitamin K‐dosed group with a complete response or partial response achieved a significantly extended median time of overall survival compared with the other patients in the vitamin K‐dosed group or the patients in the sorafenib only group (26.1 months vs 9.0 months; HR, 0.34; 95% CI: 0.11–0.95; *p* = 0.046 or 11.5 months; HR, 0.16; 95% CI: 0.034–0.70; *p* = 0.006, respectively). Dosing vitamin K could augment the anticancer action of sorafenib against HCC.

## Graphical abstract

Vitamin K dosing during sorafenib treatment against HCC showed higher ORR and prolonged PFS significantly. The vitamin K dosing might enhance the antitumor action of sorafenib by suppressing des‐γ‐carboxy prothrombin, which is a factor that promotes angiogenesis and tumor growth.

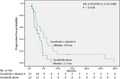

## 1 INTRODUCTION

For the past decade, sorafenib, an oral multikinase inhibitor, has been the only first‐line anticancer medicine against advanced hepatocellular carcinoma (HCC). It suppresses cancer cell proliferation and tumor angiogenesis by inhibiting c‐Raf and B‐Raf in tumor cells and the VEGF receptors of vascular endothelial cells.^1^ However, the objective response rate (ORR) of sorafenib for advanced HCC is less than 5%.^2^, ^3^

Carr et al. reported that combination treatment with sorafenib and vitamin K induced the growth inhibition and apoptosis of liver cancer cells. They also found augmented regression of transplanted tumors in rats dosed with sorafenib and vitamin K compared to rats dosed with only sorafenib.^4^, ^5^ In Japan, many cirrhotic patients with HCC were dosed with vitamin K for osteoporosis, because vitamin K sustains bone mineral density to prevent osteoporotic fracture.^6^ We retrospectively examined how vitamin K supplementation affected the outcome of sorafenib treatment in HCC patients.^7^ They found that dosing vitamin K provided some advantages regarding the sorafenib treatment outcome, possibly because of suppressing the des‐γ‐carboxy prothrombin (DCP) production of tumor cells. To confirm the advantages of the vitamin K combination treatment against advanced HCC, we conducted a phase II, randomized, open‐label trial.

## 2 METHODS

### 2.1 Patients

Patients with advanced HCC who had not received previous systemic therapy and had unresectable tumors with macroscopic vascular invasion, extrahepatic spread, or transarterial chemoembolization failure were eligible. Additionally, the eligibility criteria included an age between 18 and 85 years, an ECOG performance status score of 2 or less, a Child‐Pugh liver function score of 7 or less, a life expectancy of more than 12 weeks, a platelet count of ≥60 × 10^9^/L, a hemoglobin level of ≥8.5/dL, a prothrombin international normalized ratio of ≤2.3, an albumin concentration of ≥2.8 g/dL, a total bilirubin level ≤3.0 mg/dL, an alanine aminotransferase level of ≤5 times the upper limit of the normal range, and a serum creatinine concentration of ≤1.5 times the upper limit of the normal range. The exclusion criteria included serious infections, history of locoregional therapy, and gastrointestinal bleeding up to 30 days before study entry. It was required that patients possessed at least one target lesion to be evaluated by the modified Response Evaluation Criteria in Solid Tumors (RECIST).^8^

All patients provided written informed consent before enrollment in the study. The study protocol conformed to the guidelines of the Declaration of Helsinki and local laws, and it was also approved by the ethics committee of Osaka General Medical Center.

### 2.2 Study procedures

This phase 2, randomized, open‐label study was conducted at Osaka General Medical Center, Osaka, Japan. All eligible patients were randomly assigned at a 1:1 ratio by a computer‐generated randomization scheme to receive sorafenib (Nexavar, Bayer Healthcare Pharmaceuticals, Leverkusen, Germany) + vitamin K2 (menatetrenone: Glakay, Eisai Co., Ltd., Tokyo, Japan) or sorafenib only. Sorafenib was given at 400 mg twice daily, and vitamin K was given orally at 15 mg three times daily. Sorafenib dosing interruption or dose reduction was performed according to the prescribing information. The treatment was continued until radiological disease progression, unacceptable adverse events, a decline to performance status 3 or 4, or death. Locoregional treatment (transarterial chemoembolization, radiofrequency ablation, or radiation) or systemic chemotherapy instead of sorafenib was allowed after ceasing treatment with sorafenib + vitamin K or sorafenib only.

### 2.3 Assessments

The primary outcome of the study was progression‐free survival (PFS). PFS was assessed from the date of randomization until disease progression defined by radiological examination or death. In this study, we considered that the PFS was suitable to directly assess the efficacy of vitamin K combination dosing, because overall survival (OS) could be influenced by some treatments against HCC after ceasing sorafenib or sorafenib + vitamin K combination. All patients underwent a computed tomography or magnetic resonance imaging examination with dynamic contrast enhancement within 2 weeks before the beginning of the treatment and every 4 to 6 weeks after the beginning of the treatment until tumor progression. The radiological response was evaluated according to the modified RECIST by independent review (a radiologist and a hepatologist). The ORR is the percentage of patients who obtained a complete response (CR) or a partial response (PR) that was sustained for at least 4 weeks. The disease control rate was defined as the maintenance of a CR, a PR or stable disease (SD) for at least 4 weeks.

The secondary outcomes were OS and safety. OS was defined as the time from the date of randomization until the date of death from any cause. Safety was assessed according to the Common Terminology Criteria for Adverse Events, version 4.0.

The DCP level inn serum was measured by ECLIA (Wako Pure Chemical Industries, Ltd., Osaka, Japan). The DCP and α‐fetoprotein (AFP) levels in serum were tested every 4 weeks. Patients were examined every 2 to 4 weeks for compliance and safety assessments. They reported adverse events and underwent clinical laboratory tests, a physical examination and vital sign measurements.

### 2.4 Statistical analysis

We calculated the sample size based on the findings of the previous retrospective study, which suggested the enhanced antitumor outcome of sorafenib by dosing vitamin K.^7^ The median PFS was improved from 2 months in the sorafenib only group to 6 months in the vitamin K‐dosed group, and the hazard ratio (HR) was 0.25. The enrollment of 38 patients was estimated to be needed with 90% power and a two‐sided type I error of α=0.05 to detect a difference between the vitamin K‐dosed group and the sorafenib only group. Assuming an approximately 15% loss to follow‐up rate, we planned to enroll 44 patients, 22 for the vitamin K‐dosed versus 22 for the sorafenib only. Interim analysis was not performed. The Kaplan‐Meier method and log‐rank test were used to estimate the PFS and OS of the two groups. The Student t‐test, Mann‐Whitney U test, chi‐square test and Fisher exact test, were used for analyzing the baseline characteristics, ORR, disease control rates and adverse events. The paired t‐test was performed for the logarithmically transformed DCP and AFP values. All P values reported are two‐sided, and *p* < 0.05 was considered statistically significant.

## 3 RESULTS

### 3.1 Patients

**FIGURE 1:**
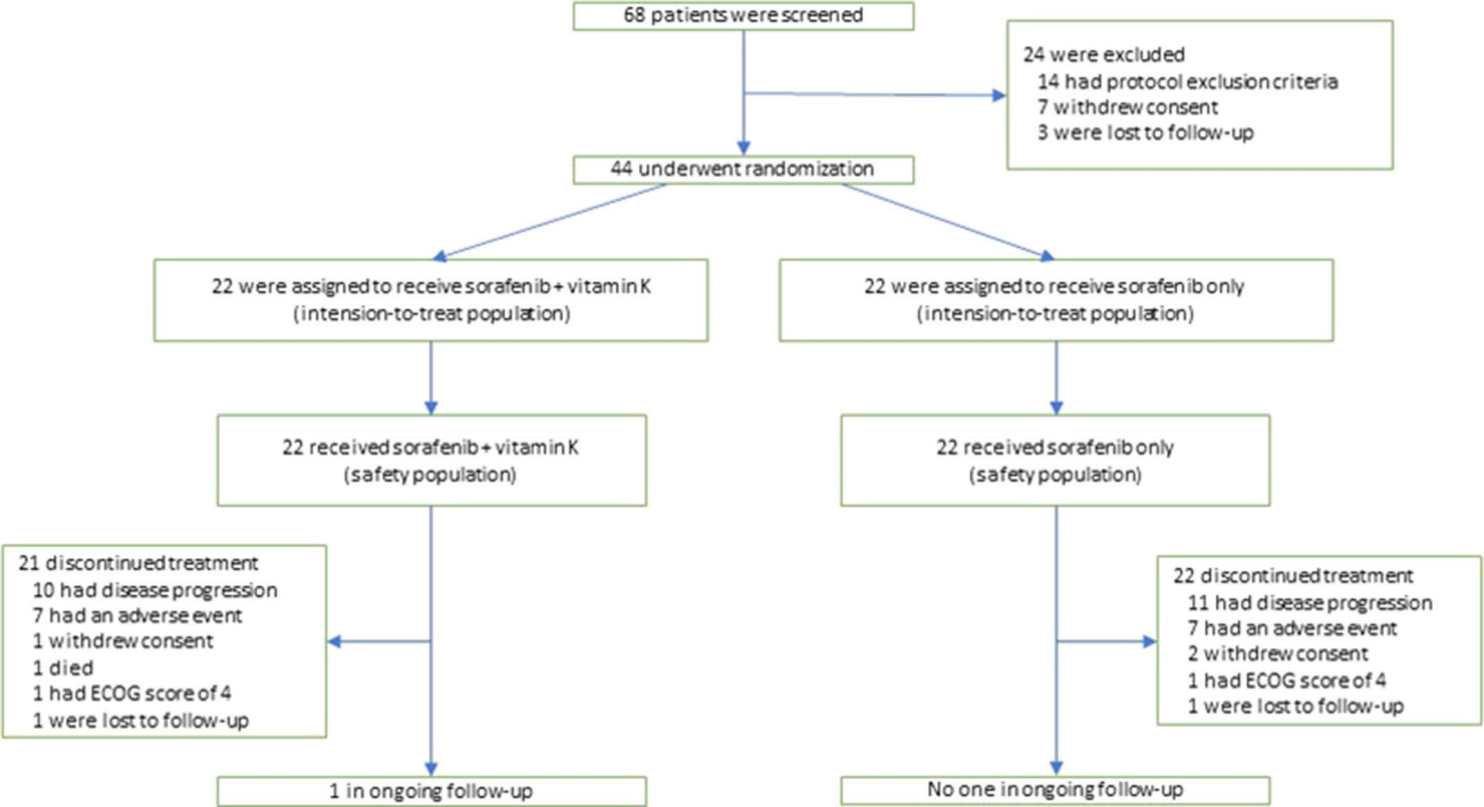
Trial profile. ECOG: Eastern Cooperative Oncology Group

From 1 May 2012, to 1 May 2016, 68 patients treated at Osaka General Medical Center were screened. Twenty‐four patients were excluded; 14 met the protocol exclusion criteria, 7 withdrew consent, and 3 were lost to follow‐up. The remaining 44 patients underwent randomization. Twenty‐two were assigned to the vitamin K‐dosed group, and 22 were assigned to the sorafenib only group. Intension‐to‐treat analysis was performed for the 44 patients (Figure 1). There were no relevant differences between the two groups in the baseline characteristics, age, sex, ECOG performance, cause of liver disease, Barcelona Clinic Liver Cancer (BCLC) stage, macroscopic vascular invasion, extrahepatic spread, Child‐Pugh status or serum AFP level (Table 1). Most patients had a good performance status (PS‐0) (82%) and well‐preserved liver function (Child‐Pugh classification A) (84%). HCV infection was the predominant cause of background liver disease (68%). Sixty‐six percent of the patients had advanced HCC (BCLC stage C). The cutoff date for evaluation in the study was 1 November 2018.

**TABLE 1:** Basic characteristics of the patients

Variables	Sorafenib +vitamin K	Sorafenib alone	P value
Age, mean ±SD	72.±8.3	71.9 ± 8.9	0.77
Sex, n (%)			
Male	17 (77)	18 (82)	0.50
Female	5 (23)	4 (18)	
ECOG performance status, n (%)			
0	18 (82)	18 (82)	0.65
1	4 (18)	4 (18)	
Cause of disease, n (%)			
HCV infection	17 (77)	13 (59)	0.74
HBV infection	2 (9)	5 (23)	
Alcohol	2 (9)	3 (14)	
Other	1 (5)	1 (5)	
BCLC stage, n (%)			
B	6 (27)	9 (41)	0.34
C	16 (73)	13 (59)	
Macroscopic vascular invasion, n (%)	9 (41)	6 (27)	0.34
Extraheparic spread, n (%)	9 (41)	11 (50)	0.55
Child‐Pugh status, n (%)			
A	19 (86)	18 (82)	0.50
B	3 (14)	4 (18)	
AFP (ng/ml)			
Median	92.6	76.8	0.40
Range	1.9–105146.7	1.6–133133.9	

AFP, α‐fetoprotein; BCLC, Barcelona Clinic Liver Cancer; HBV, hepatitis B virus; HCV, hepatitis C virus.

### 3.2 Efficacy

**FIGURE 2:**
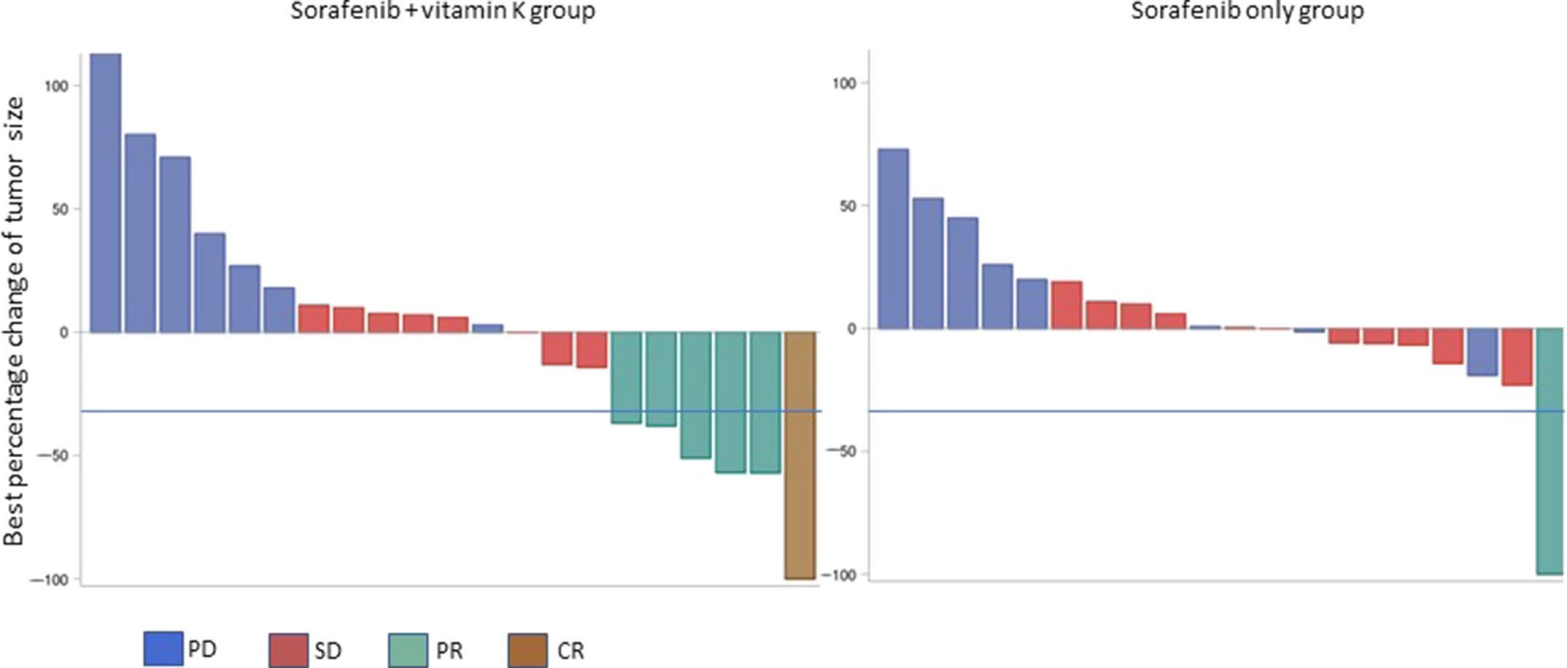
Best percent change in tumor size evaluated by modified RECIST. Six of the 8 patients with tumor size reduction achieved a CR or PR in the vitamin K‐dosed group, whereas only one of 7 patients with tumor size reduction showed a PR in the sorafenib only group. Blue lines show a 30% reduction. One patient in the vitamin K‐dosed group and 2 in the sorafenib only group were excluded due to lack of assessment

In the vitamin K‐dosed group, one patient obtained a CR; 5, PR; 8, SD; and 8, progressive disease (PD). In the sorafenib only group, no patients achieved a CR, and one patient showed a PR, 11 showed SD, and 10 showed PD. The ORR in the vitamin K‐dosed group was significantly higher than that in the sorafenib only group (27.3% vs 4.5%, respectively; *p* = 0.039). There was no significant difference in the disease control rate between the two groups (63.6% vs 54.5%, respectively; *p* = 0.54). Waterfall analysis showed the tumor size reduction in approximately 40% of patients in the vitamin K‐dosed group, which was similar in the sorafenib only group. However, 75% of the patients with tumor size reduction achieved a CR or PR in the vitamin K‐dosed group, whereas only 13% of the patients with tumor size reduction showed a PR in the sorafenib only group (*p* = 0.020) (Figure 2).

**FIGURE 3:**
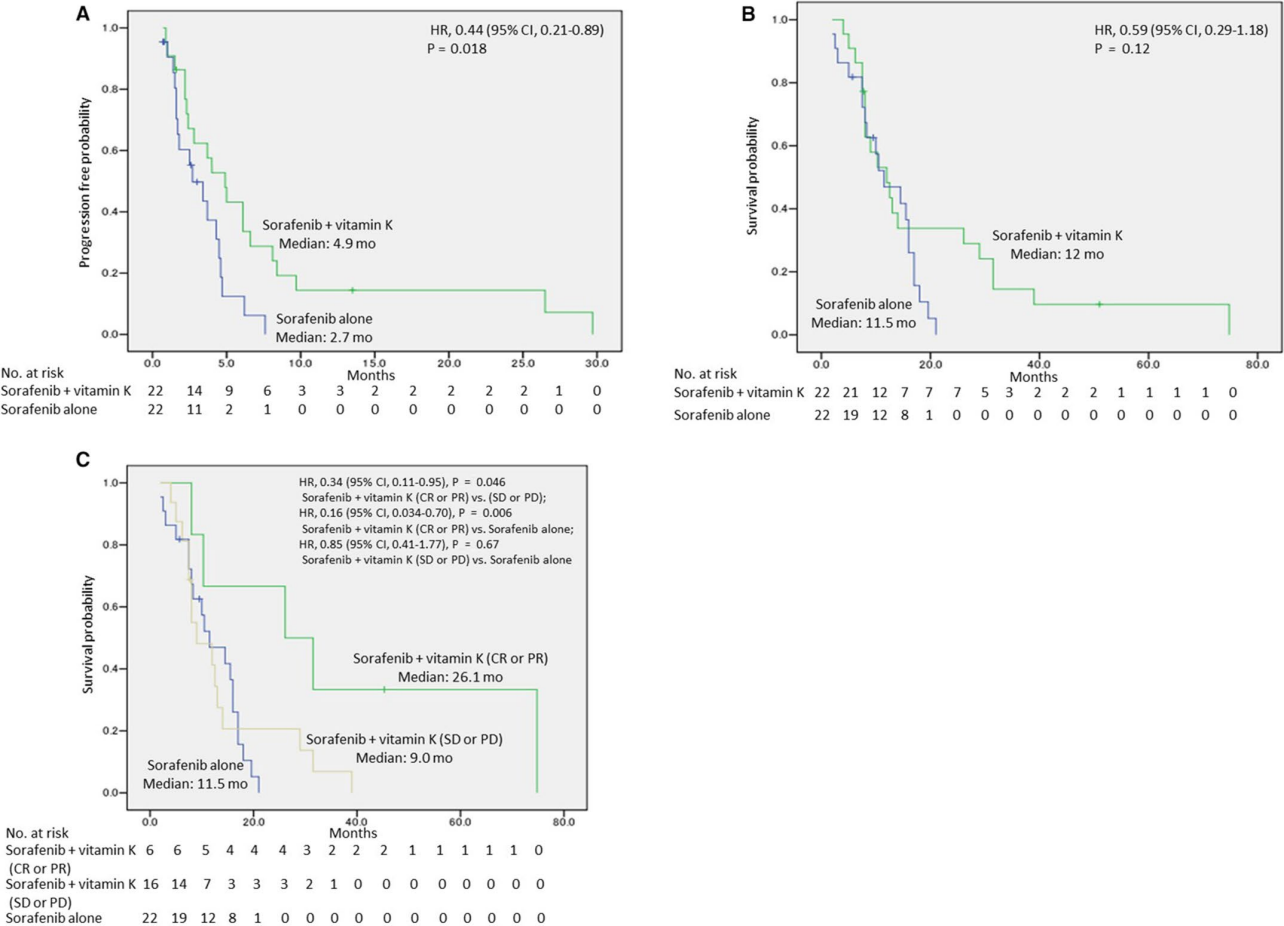
Kaplan‐Meier curves of progression‐free survival (A), overall survival (B), and overall survival of patients with a CR or PR in the vitamin K‐dosed group, patients with SD or PD in the vitamin K‐dosed group, and patients in the sorafenib only group (C)

The median PFS was significantly longer in the vitamin K‐dosed group than in the sorafenib only group (4.9 months vs 2.7 months, respectively; HR, 0.44; 95% confidence interval (CI): 0.21–0.89; *p* = 0.018) (Figure 3A). The median OS was 12.0 months in the vitamin K‐dosed group and 11.5 months in the sorafenib only group (HR, 0.59; 95% CI: 0.29–1.18; *p* = 0.12). The Kaplan–Meier curve of OS showed some long‐term survivors in the vitamin K‐dosed group. The 2‐ and 3‐year survival rates were 34% and 15% in the vitamin K‐dosed group, while both were 0% in the sorafenib only group (Figure 3B). To examine the attributes of long‐term survivors in the vitamin K‐dosed group, a Cox proportional hazards model was used to assess the involvement of baseline factors, including age, ECOG performance status, Child‐Pugh status, serum AFP level, macroscopic vascular invasion, extrahepatic spread, and the response to treatment (CR +PR vs SD +PD). Only the response to the treatment was a significant factor contributing to OS in the vitamin K‐dosed group (HR, 0.069; 95% CI: 0.009–0.53; *p* = 0.01). Figure 3C shows the Kaplan–Meier curves of OS for patients with a CR or PR in the vitamin K‐dosed group, patients with SD or PD in the vitamin K‐dosed group, and all patients in the sorafenib only group (median OS, 26.1 months, 9.0 months, and 11.5 months, respectively). In the sorafenib only group, only one patient obtained a PR, and his survival time was 8.0 months. Thus, we compared all patients in the sorafenib only group, including the patient with a PR, with those in the other two groups. The patients with a CR or PR in the vitamin K‐dosed group had significantly prolonged OS compared with the patients with SD or PD in the vitamin K‐dosed group or all patients in the sorafenib only group (HR, 0.34; 95% CI: 0.11–0.95; *p* = 0.046 or HR, 0.16; 95% CI: 0.034–0.70; *p* = 0.006, respectively). No significant difference in OS was found between patients with SD or PD in the vitamin K‐dosed group and all patients in the sorafenib only group (HR, 0.85; 95% CI: 0.41–1.77; *p* = 0.67).

### 3.3 Changes of serum DCP and AFP levels during treatment

#### 3.3.1 Serum DCP and AFP levels before and 8 weeks after starting sorafenib +vitamin K combination treatment

**FIGURE 4:**
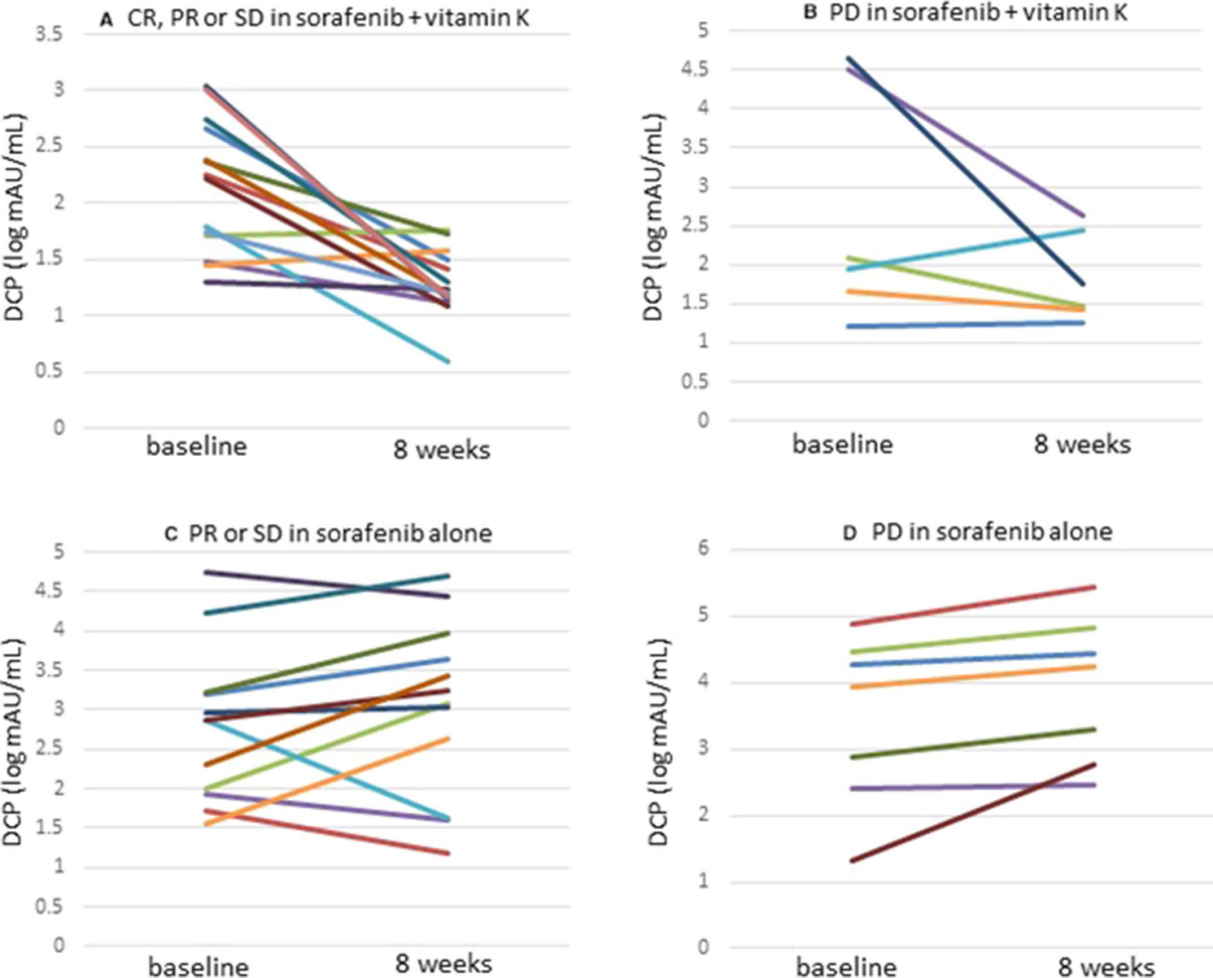
Changes in serum DCP levels before and 8 weeks after starting sorafenib +vitamin K or sorafenib alone dosing. (A) Patients with a CR, a PR or SD in vitamin K‐dosed group, (B) Patients with PD in vitamin K‐dosed group, (C) Patients with a PR or SD in sorafenib only group, (D) Patients with PD in sorafenib only group

**FIGURE 5:**
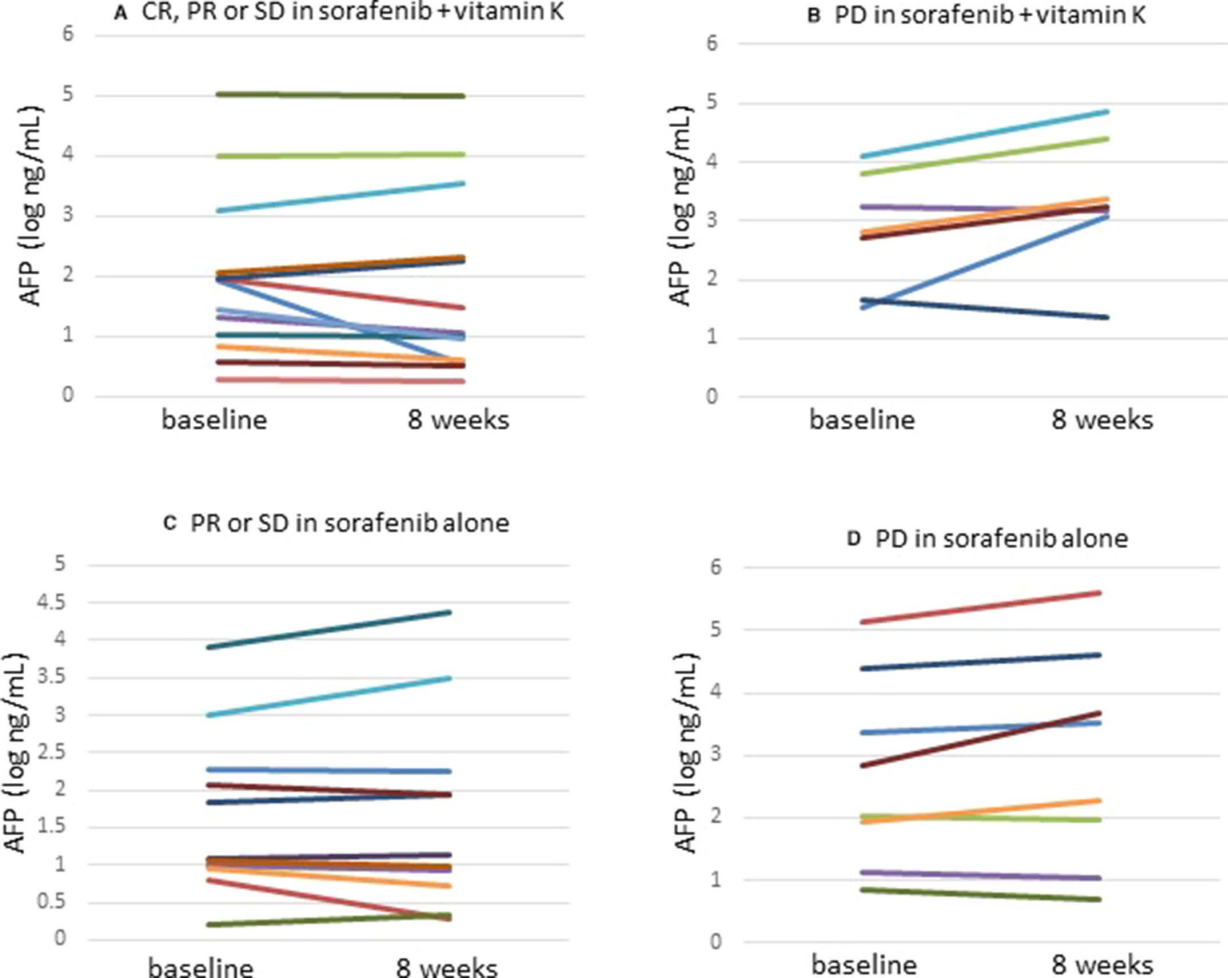
Changes in serum AFP levels before and 8 weeks after starting sorafenib +vitamin K or sorafenib alone dosing. (A) Patients with a CR, a PR or SD in vitamin K‐dosed group, (B) Patients with PD in vitamin K‐dosed group, (C) Patients with a PR or SD in sorafenib only group, (D) Patients with PD in sorafenib only group

In patients with a CR, a PR or SD, the serum DCP level markedly declined (mean ± SD (log mAU/mL): 2.15 ± 0.58 to 1.29 ± 0.30; *p* < 0.001), while the serum AFP level tended to decrease (mean ± SD (log ng/mL): 1.95 ± 1.37 to 1.81 ± 1.52; *p* = 0.28) (Figures 4A, 5A). In patients with PD, the serum DCP level also tended to decrease (mean ± SD (log mAU/mL): 2.85 ± 1.45 to 1.83 ± 0.53; *p* = 0.079), although the serum AFP level tended to increase (mean ± SD (log ng/mL): 2.66 ± 0.98 to 3.17 ± 1.11; *p* = 0.11) (Figures 4B, 5B).

#### 3.3.2 Serum DCP and AFP levels before and 8 weeks after starting sorafenib only treatment

In the patients with a PR or SD, the serum DCP level tended to increase (mean±SD (log mAU/mL): 2.80 ± 0.98 to 3.05 ± 1.12; *p* = 0.27), whereas the serum AFP level did not change (mean ± SD (log ng/mL): 1.66 ± 1.09 to 1.67 ± 1.30; *p* = 0.85) (Figures 4C, 5C). In patients with PD, the serum DCP level significantly increased (mean ± SD (log mAU/mL): 3.45 ± 1.28 to 3.92 ± 1.11; *p* = 0.034), while the serum AFP level tended to increase (mean ± SD (log ng/mL): 2.70 ± 1.52 to 2.92 ± 1.73; *p* = 0.11) (Figures 4D, 5D).

### 3.4 Safety

There was no relevant difference in the overall incidence of treatment‐related adverse events between the vitamin K‐dosed group and the sorafenib only group (91% vs 91% for any grade and 59% vs 64% for grade 3, respectively). No drug‐related deaths or grade 4 adverse events were observed in the study. Adverse events which occurred in at least one patient in either group were shown in Table 2.

**TABLE 2:** Incidence (percent) of drug‐related adverse events

Adverse event	Sorafenib + vitamik K		Sorafenib + vitamik K		*P* value	
	Any grade	Grade 3	Any grade	Grade 3	Any grade	Grade 3
Overall incidence	91	59	91	64	0.50	0.55
Palmar‐plantar erythodysaesthesia	27	9	45	27	0.21	0.12
Aspantate aminotransferase increase	32	9	18	14	0.30	0.50
Rash/desquamation	32	5	14	9	0.15	0.50
Hypophosphatemia	32	9	5	5	0.02	0.50
Pancreatic amylase increase	23	14	23	9	1.00	0.50
Diarrhea	18	5	9	5	0.33	0.76
Fatigue	18	5	9	5	0.33	0.76
Hypertension	18	0	27	5	0.47	0.50
Hypothyroidism	14	0	0	0	0.12	NA
Dysphonia	9	0	14	0	0.50	NA
Alopecia	5	0	5	0	0.76	NA
Gastrointestinal hemorrhage	0	0	14	5	0.12	0.50

## 4 DISCUSSION

In this study, sorafenib + vitamin K combination treatment showed a much higher ORR (27.3%) than sorafenib only (4.5%). The median time of PFS was extended in the vitamin K‐dosed group compared with the sorafenib only group (4.9 months vs 2.7 months). Waterfall analysis showed that approximately 40% of patients obtained tumor size reduction in the vitamin K‐dosed group, similar to the sorafenib only group. However, 75% of patients with tumor size reduction achieved a CR or PR in the vitamin K‐dosed group, although only 13% of patients with tumor size reduction showed a PR in the sorafenib only group. This suggests that vitamin K itself might not deteriorate tumor growth and that vitamin K could enhance antitumor effects through cooperation with sorafenib. Vitamin K and sorafenib seem to be a good combination for achieving antitumor effects. We found some long‐term survivors in the vitamin K‐dosed group, although there was no significant difference in OS between the two groups. Response analysis revealed that the OS of responders to the vitamin K combination treatment was much more prolonged than that of the non‐responders or patients treated with sorafenib only. These findings suggest that patients could achieve very long‐term survival upon responding to the vitamin K combination treatment.

For a decade, sorafenib had been the only first‐line anticancer medicine for systemic chemotherapy against advanced HCC; then, lenvatinib, an oral multi‐kinase inhibitor, became available.^9^ The phase 3 study of lenvatinib versus sorafenib showed that lenvatinib was superior to sorafenib in terms of the ORR and PFS.^10^ Although no significant difference in OS was observed between lenvatinib and sorafenib, lenvatinib has been replacing sorafenib as the first‐line medicine for systemic chemotherapy against HCC because of its higher ORR and longer PFS.^11^ The ORR of sorafenib + vitamin K (27.3%) in this study is equal to that of lenvatinib (24.1%) reported by Kudo et al.^10^ Furthermore, responders to vitamin K combination treatment might achieve very long‐term survival. Although this was a pilot study conducted at a single center, the findings suggest that sorafenib might remain the first‐line medicine in combination with vitamin K dosing.

As an exploratory analysis, we focused on changes in the DCP level during treatment. The serum DCP level drastically declined after vitamin K combination treatment, even in patients with PD. In contrast, the DCP level increased after treatment with sorafenib only, even in patients with a PR or SD. These findings show that dosing vitamin K strongly suppressed the elevation in the DCP level caused by sorafenib treatment. Deteriorated tumor angiogenesis by sorafenib leads to tumor cell ischemia. Murata et al. reported that ischemic conditions disturb the transportation of vitamin K into tumor cells and decrease the activity of γ‐glutamyl carboxylase, which is a vitamin K‐dependent enzyme that transforms DCP into prothrombin.^12^, ^13^ Thus, sorafenib treatment could lead to the accumulation of DCP in tumor cells and increase the serum DCP level. DCP is known as a paracrine tumor angiogenesis factor and an autocrine tumor growth factor.^14^, ^15^ Therefore, the DCP elevation caused by sorafenib treatment seems to allow viable ischemic tumor cells to survive and grow under anticancer treatment. With high dosing of vitamin K, vitamin K is transferred into ischemic tumor cells and decreases DCP production.^12^ Suppression of the DCP level by dosing vitamin K might enhance the anticancer effect of sorafenib.^16^

Vitamin K2 (Glakay) is a safe and economical medicine. The price of the dose for one day (45 mg) is 81.3 Japanese yens (approximately 0.7 USD).

To the best of our knowledge, this study is the first prospective trial showing the efficacy and safety of dosing vitamin K during sorafenib treatment against HCC. More studies are needed to confirm the advantage of the sorafenib + vitamin K combination, considering possible various options of future HCC treatments, including other tyrosine‐kinase inhibitors and immune checkpoint inhibitors.

## CONFLICT OF INTEREST

The authors have no conflicts of interest to declare.

## AUTHOR CONTRIBUTIONS

Yoshimichi Haruna and Seiichi Kawamoto contributed to conception and design. Yoshimichi Haruna, Takayuki Yakushijin, and Seiichi Kawamoto contributed to collection and assembly of data. Yoshimichi Haruna, Takayuki Yakushijin, and Seiichi Kawamoto contributed to analysis and interpretation of data. Yoshimichi Haruna and Seiichi Kawamoto contributed to drafting of the manuscript. All authors contributed to the approval of the final version of the manuscript.

## Data Availability

Raw data were generated at Osaka General Medical Center. Derived data supporting the findings of this study are available from the corresponding author Y.H. on request.
